# Laparoscopic versus open emergency repair for strangulated abdominal wall hernias: a propensity-score-matched retrospective cohort study

**DOI:** 10.3389/fsurg.2026.1949053

**Published:** 2026-09-09

**Authors:** Ahmet Topcu, İsmail Ege Subaşı, Furkan Saydin

**Affiliations:** Department of General Surgery, Sancaktepe Şehit Prof. Dr. İlhan Varank Training and Research Hospital, Istanbul, Türkiye

**Keywords:** emergency hernia, incarcerated hernia, laparoscopic repair, morbidity, mortality, open repair, strangulated hernia, wound complications

## Abstract

**Background and objectives:**

The optimal surgical approach for incarcerated or strangulated abdominal wall hernias remains debated. While laparoscopic techniques are well established electively, evidence on their emergency-setting role is limited. This study compared open and laparoscopic repair in patients undergoing emergency surgery for incarcerated or strangulated abdominal wall hernias, focusing on postoperative morbidity, mortality, operative time, and hospital stay.

**Materials and methods:**

This retrospective single-center cohort study included 180 consecutive patients treated between January 2023 and June 2025, analyzed by initially intended approach (open, *n* = 90; laparoscopic, *n* = 90; converted patients retained in their original group). Demographics, hernia type, intraoperative findings, complications, mortality, and length of stay were compared using Fisher's exact and Mann–Whitney U tests. Wound-related morbidity (surgical site infection and/or seroma) was analyzed as a composite outcome via multivariate logistic regression adjusted for age, sex, BMI, ASA class, hernia type, smoking, symptom duration, contamination, and bowel resection, with a reduced four-covariate sensitivity model. Propensity-score matching, subgroup analyses by hernia type, and mesh-use/no-bowel-resection sensitivity analyses were also performed; all were prespecified but exploratory, without correction for multiple testing.

**Results:**

Groups were comparable in baseline characteristics (all *p* > 0.05), with no postoperative deaths. Operative time was longer with laparoscopy (82.0 ± 16.7 vs. 76.0 ± 13.5 min, *p* = 0.025), while hospital stay was shorter (3.9 ± 1.0 vs. 5.2 ± 0.9 days, *p* < 0.001) and postoperative ileus less frequent (1.1% vs. 8.9%, *p* = 0.034). Wound complications were numerically lower after laparoscopy (13.3% vs. 22.2%) but not statistically significant (*p* = 0.172). No covariate independently predicted wound morbidity on multivariate analysis (technique OR 0.59, 95% CI 0.26–1.33, *p* = 0.203). Propensity-matched analysis (68 pairs) confirmed the operative-time and length-of-stay findings; the length-of-stay benefit was consistent across all hernia-type subgroups and sensitivity analyses.

**Conclusions:**

Laparoscopic repair of strangulated abdominal wall hernias was associated with shorter hospital stay and lower ileus rates, but longer operative time, than open repair, with no significant difference in wound morbidity or mortality. Given the non-randomized design and limited power for the wound-complication comparison (∼35%), these findings—particularly regarding wound morbidity—should be considered hypothesis-generating. Laparoscopic repair may be a reasonable option in carefully selected, hemodynamically stable patients, pending confirmation by adequately powered multicenter trials.

## 1 Introduction

Abdominal wall hernias are among the most frequently encountered conditions in general surgical practice and include inguinal, umbilical, incisional, epigastric, and femoral hernias. Inguinal hernias represent the most common subtype, accounting for approximately 70%–75% of all abdominal wall hernias and a substantial proportion of routine elective surgical workload (1, 2).

Although most abdominal wall hernias follow a benign course, a subset present acutely with incarceration or strangulation. These complications can progress to bowel ischemia and necrosis, and emergency surgical intervention is mandatory to reduce the risk of associated morbidity and mortality (3).

The management of inguinal hernias differs from that of other abdominal wall hernias in terms of anatomical complexity and surgical strategy. While inguinal hernia repair is largely standardized, incisional and umbilical hernias frequently require more individualized surgical planning, particularly when repaired under emergency conditions (4, 5).

Both open and laparoscopic techniques are used for hernia repair. Open repair, exemplified by the Lichtenstein technique, has traditionally been regarded as the standard of care (4). Since the introduction of minimally invasive approaches, laparoscopic techniques — including transabdominal preperitoneal (TAPP), totally extraperitoneal (TEP), and extended totally extraperitoneal (eTEP) repair — have become increasingly accepted, primarily in the elective setting (6).

A substantial body of literature, largely derived from elective surgery, has reported that laparoscopic repair is associated with less postoperative pain, faster recovery, and earlier return to normal activity compared with open repair (7–9). Meta-analyses and large registry-based studies have further corroborated these findings, demonstrating lower wound complication rates and shorter hospital stay with laparoscopic approaches (10–12).

Evidence specific to the emergency setting is considerably less mature. In strangulated or incarcerated hernias — particularly when bowel resection may be required — open surgery continues to be favored by many surgeons because of concerns about intraoperative bowel viability assessment, exposure, and operative time under laparoscopy (13–15). Existing comparative series in this population are generally small, single-center, and susceptible to selection bias, since laparoscopy is typically reserved for hemodynamically stable patients presenting earlier in the course of their disease. This selection pattern makes it difficult to separate the effect of the surgical technique itself from the effect of case selection, a limitation that also applies to the present study, as detailed in the Discussion.

Given this gap, the present study was designed to compare open and laparoscopic repair in a relatively large, size-balanced single-center cohort of patients undergoing emergency surgery for incarcerated or strangulated abdominal wall hernia, focusing on postoperative morbidity, mortality, operative time, and length of hospital stay, while explicitly accounting for baseline differences through multivariate adjustment.

## 2 Materials and methods

### 2.1 Study design and population

**Figure 1:**
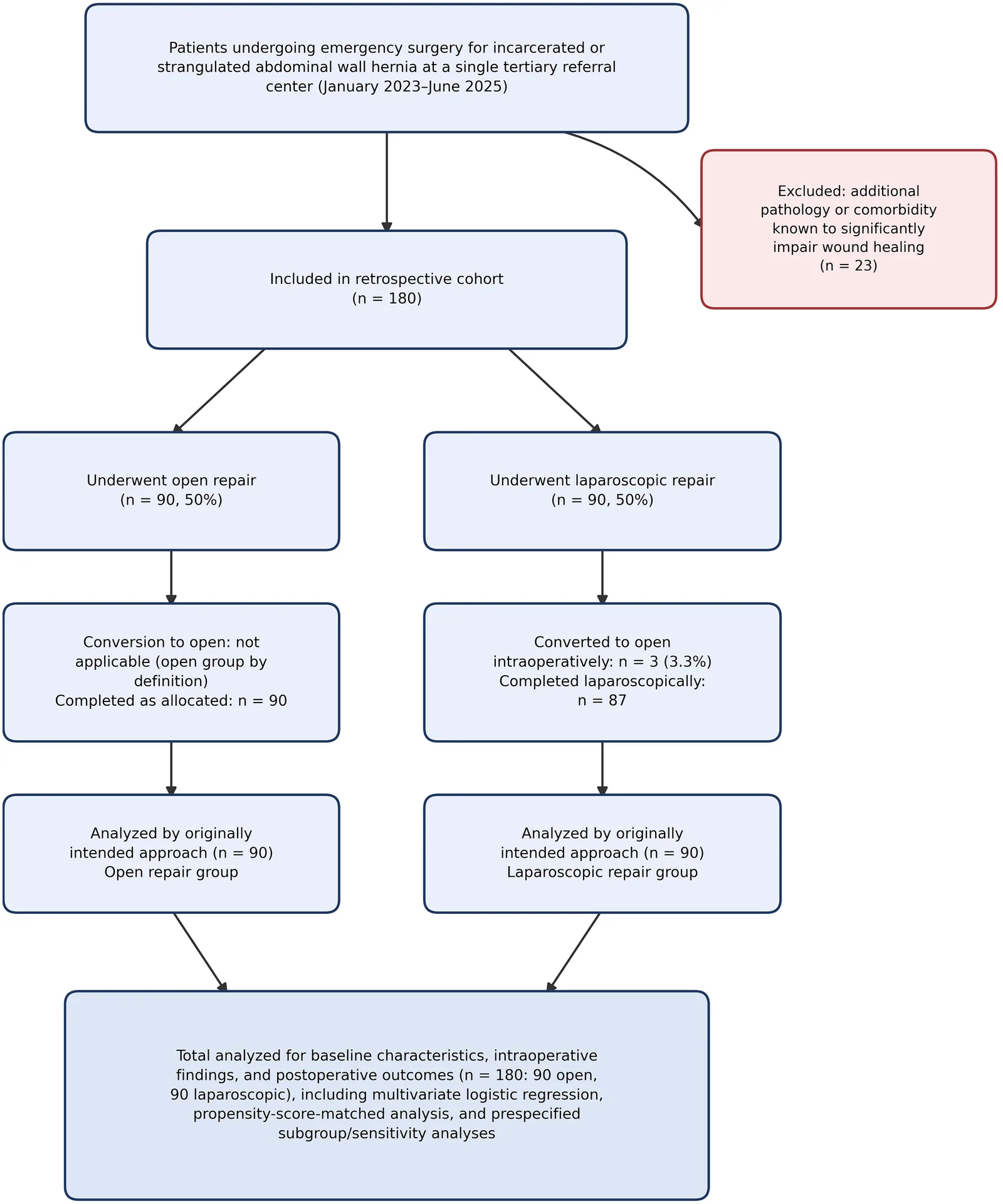
STROBE patient flow diagram, showing inclusion, allocation to open (*n* = 90) or laparoscopic (*n* = 90) repair analyzed according to the originally intended approach, intraoperative conversion (3 laparoscopic patients converted to open but retained in the laparoscopic group for analysis), and analysis of the full cohort (*n* = 180). Revised in this version to correct the alignment of the arrow leading to the exclusion box.

This retrospective, single-center cohort study included 180 patients (104 women, 76 men; mean age 63.5 ± 13.8 years) who underwent emergency surgery for strangulated abdominal wall hernia at our tertiary referral center between January 2023 and June 2025 (29-month recruitment period). Of 203 patients assessed for eligibility during the study period, 23 (11.3%) were excluded because of additional pathology or comorbidities known to significantly impair wound healing, leaving 180 patients for analysis. The study is reported in accordance with the STROBE (Strengthening the Reporting of Observational Studies in Epidemiology) guideline for cohort studies; the patient flow through inclusion, allocation, and analysis is summarized in Figure 1, and a completed STROBE checklist is provided as a Supplementary File.

Patients were divided into two equally sized groups according to the surgical technique initially intended/attempted (see Section 2.2 regarding conversions): open repair (*n* = 90, 50%) and laparoscopic repair (*n* = 90, 50%). The decision between open and laparoscopic approach was made by the operating surgical team based on hemodynamic stability, clinical and radiological suspicion of bowel compromise, hernia type and size, and surgeon experience with laparoscopic emergency repair; it was not randomized. No written institutional protocol or algorithm dictated this choice during the study period; the decision was made jointly by the on-call attending and operating team at the time of surgery, informed by hemodynamic parameters, CT findings suggestive of bowel compromise (e.g., free fluid, pneumatosis, or reduced bowel-wall enhancement), hernia type/size, and which surgeons experienced in laparoscopic emergency repair were available on that shift. As shown in Table 1, the two groups were closely balanced across all measured baseline characteristics, including symptom duration before admission, with no statistically significant differences observed. Unlike some previously reported single-center series, the two groups were of equal size in this cohort, which reduces (though does not eliminate) the loss of precision that a marked size imbalance would otherwise introduce into between-group comparisons. This non-random allocation nonetheless remains a potential source of confounding and is discussed further below.

**Table 1:** Baseline demographic and clinical characteristics.

Variable	Open (*n* = 90)	Laparoscopic (*n* = 90)	*p*-value
Age (years), mean ± SD	62.5 ± 14.2	64.4 ± 13.4	0.344
Sex, female, *n* (%)	54 (60.0%)	50 (55.6%)	0.651
BMI (kg/m^2^), mean ± SD	27.4 ± 3.7	27.1 ± 3.8	0.706
Diabetes mellitus, *n* (%)	21 (23.3%)	23 (25.6%)	0.862
Hypertension, *n* (%)	48 (53.3%)	44 (48.9%)	0.655
COPD, *n* (%)	13 (14.4%)	14 (15.6%)	1.000
Coronary artery disease, *n* (%)	16 (17.8%)	15 (16.7%)	1.000
Current smoker, *n* (%)	20 (22.2%)	32 (35.6%)	0.070
ASA score I–II, *n* (%)	53 (58.9%)	58 (64.4%)	0.540
ASA score III–IV, *n* (%)	37 (41.1%)	32 (35.6%)	0.540
Symptom duration (hours), mean ± SD	19.6 ± 11.7	19.8 ± 11.4	0.850
Symptom duration (hours), median [IQR]	17.5 [11.2–24.0]	17.0 [12.2–23.8]	0.850

Values are mean ± SD, median [IQR], or *n* (%). Mann–Whitney U test for continuous variables; Fisher's exact test for categorical variables. BMI, body mass index; ASA, American Society of Anesthesiologists; COPD, chronic obstructive pulmonary disease.

Hernia types were inguinal (*n* = 76, 42.2%), umbilical (*n* = 46, 25.6%), incisional (*n* = 42, 23.3%), and femoral (*n* = 16, 8.9%) (Table 2).

**Table 2:** Distribution of hernia types (*n* = 180).

Hernia type	n	%
Inguinal	76	42.2
Umbilical	46	25.6
Incisional	42	23.3
Femoral	16	8.9
Total	180	100

### 2.2 Surgical technique

**Figure 2:**
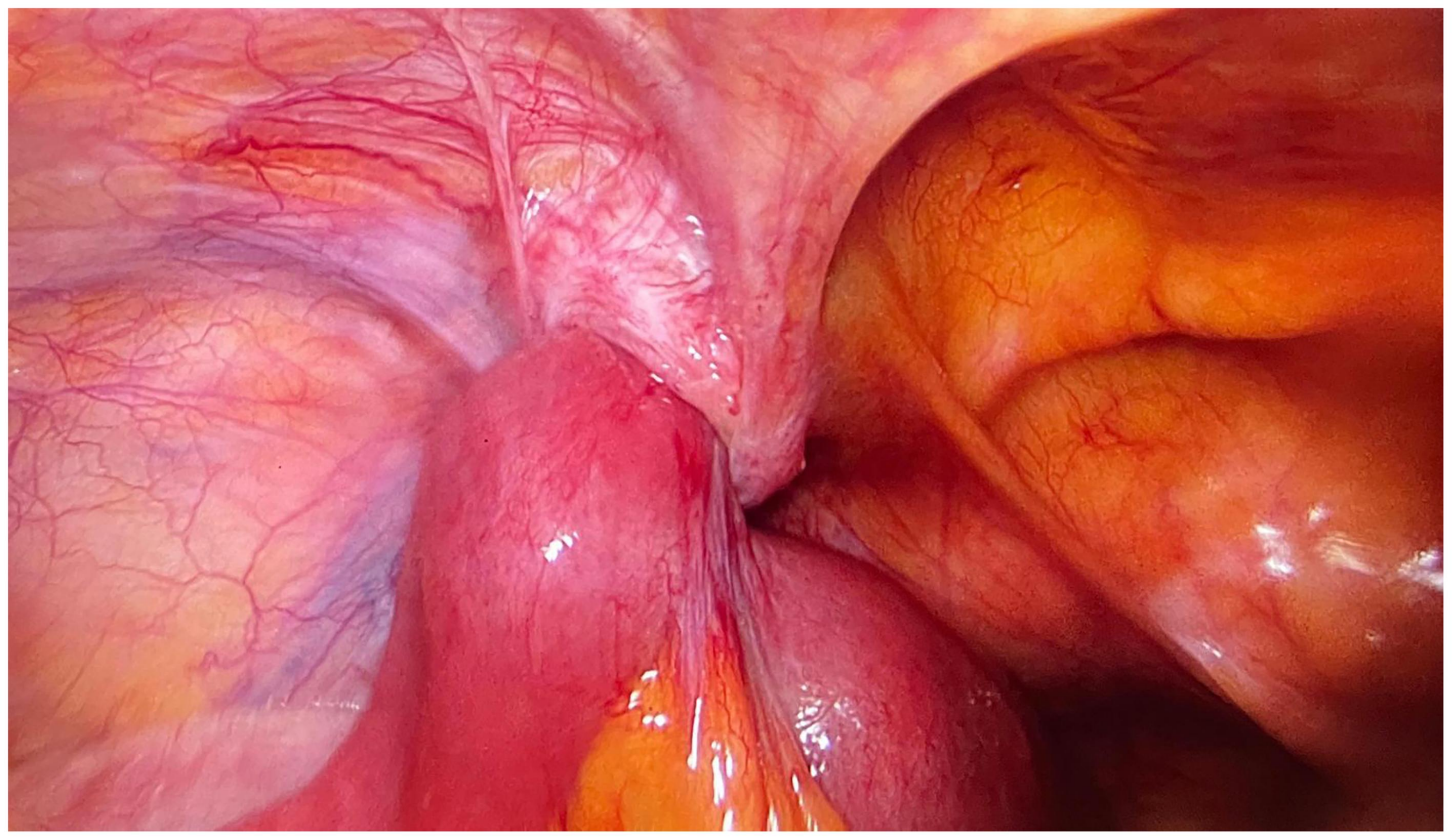
Laparoscopic intraoperative view of an incarcerated abdominal wall hernia. The herniated tissue is seen compressed at the fascial defect, with surrounding peritoneum and vascular engorgement visible. The laparoscopic approach allows direct visualization of the hernia orifice and the incarcerated contents without the need for extensive abdominal wall dissection.

**Figure 3:**
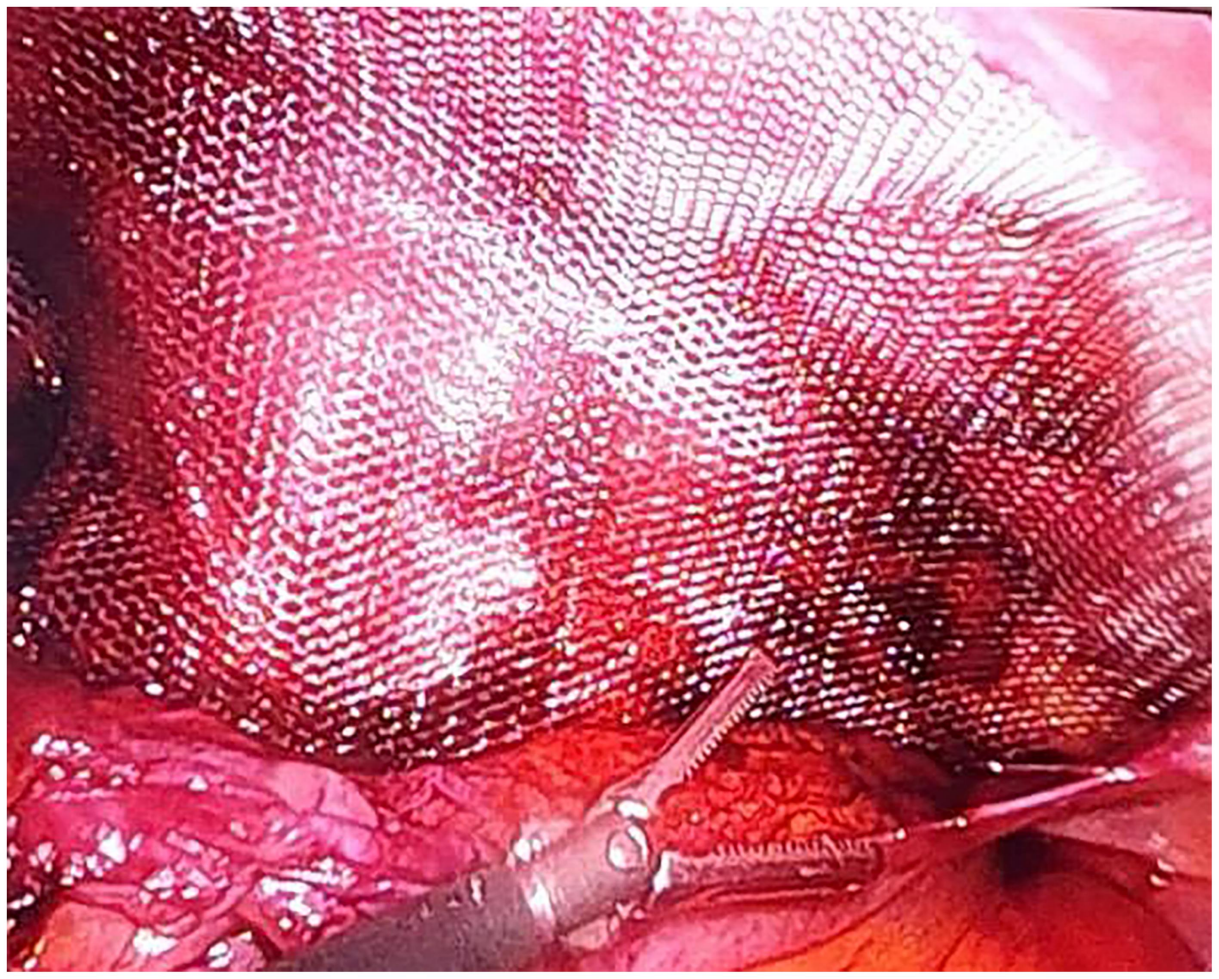
Laparoscopic view following reduction of the incarcerated hernia, demonstrating complete coverage of the fascial defect with a synthetic mesh. The mesh is positioned intraperitoneally with adequate overlap of the defect margins. Tack fixation is visible at the mesh periphery, ensuring secure anchoring to the surrounding abdominal wall.

Open repair was performed through a standard anterior approach, with primary fascial closure or mesh reinforcement according to hernia type and intraoperative findings, and with an additional intra-abdominal procedure (bowel resection) performed as required. Laparoscopic repair was performed transabdominally: after pneumoperitoneum was established, the hernia orifice and incarcerated contents were directly visualized (Figure 2), the herniated viscera were reduced, and the fascial defect was closed intraperitoneally with a synthetic mesh secured by tack fixation (Figure 3).

Conversion to an open approach was performed at the operating surgeon's discretion in cases of inadequate visualization, bowel non-viability requiring resection, or technical difficulty. Patients were analyzed according to the initially intended surgical approach (initial-attempt analysis): the 3 patients converted from laparoscopic to open surgery were retained in the laparoscopic (initially intended) group rather than reassigned to the open group, which is why Tables 1–11 and Figure 1 all report a clean 90/90 split. As this is a non-randomized retrospective cohort rather than a trial, this convention is used purely to keep the comparison groups defined consistently at the outset of surgery, not in the formal intention-to-treat sense used for randomized trials. Given the small number of conversions (3/90 laparoscopic patients, 3.3%), a *post hoc* as-treated comparison excluding these 3 patients did not materially change the operative-time or length-of-stay findings.

**Table 3:** Intraoperative findings and additional procedures.

Variable	Open (*n* = 90)	Laparoscopic (*n* = 90)	*p*-value
Strangulation, *n* (%)	28 (31.1%)	32 (35.6%)	0.635
Intraoperative bowel ischemic change (incl. reversible), *n* (%)	7 (7.8%)	8 (8.9%)	1.000
— of which, resolved without resection, n	5	5	—
— of which, resected for non-viable necrosis, n	2	3	—
Contamination, *n* (%)	5 (5.6%)	11 (12.2%)	0.189
Bowel resection (all indications), *n* (%)	4 (4.4%)	5 (5.6%)	1.000
— of which, for reasons other than necrosis (e.g., bowel-wall injury during reduction), n	2	2	—
Conversion to open, *n* (%)	0 (0%)	3 (3.3%)	0.246
Mesh used, *n* (%)	70 (77.8%)	71 (78.9%)	1.000

Values are *n* (%). Fisher's exact test was used for all comparisons; rows without a comparator group are descriptive only. “Conversion to open” is applicable only to the laparoscopic group; the open-group value of 0 (0%) is shown for completeness.

**Table 4:** Postoperative outcomes.

Outcome	Open (*n* = 90)	Laparoscopic (*n* = 90)	*p*-value
Operation time (min), mean ± SD	76.0 ± 13.5	82.0 ± 16.7	0.025
Length of hospital stay (days), mean ± SD	5.2 ± 0.9	3.9 ± 1.0	<0.001
Length of hospital stay (days), median [IQR]	5.0 [4.6–5.5]	3.8 [3.1–4.4]	<0.001
Surgical site infection, *n* (%)	12 (13.3%)	6 (6.7%)	0.213
Seroma, *n* (%)	10 (11.1%)	7 (7.8%)	0.611
Wound complication (SSI and/or seroma), *n* (%)	20 (22.2%)	12 (13.3%)	0.172
Postoperative ileus, *n* (%)	8 (8.9%)	1 (1.1%)	0.034
Reoperation within 30 days, *n* (%)	1 (1.1%)	3 (3.3%)	0.621
30-day mortality, *n* (%)	0 (0%)	0 (0%)	NA
Recurrence during follow-up, *n* (%)	4 (4.4%)	5 (5.6%)	1.000
Follow-up (months), mean ± SD	18.9 ± 5.3	18.4 ± 5.2	0.754

Values are mean ± SD, median [IQR], or *n* (%). Mann–Whitney U test for continuous variables; Fisher's exact test for categorical variables. SSI, surgical site infection. NA, not applicable (zero events in both groups precluded statistical testing of mortality).

**Table 5:** Distribution of maximum clavien-dindo complication grade.

Approach	Grade 0	Grade I	Grade II	Grade IIIa	Grade IIIb/IVa
Open (*n* = 90)	61 (67.8%)	8 (8.9%)	17 (18.9%)	3 (3.3%)	1 (1.1%)
Laparoscopic (*n* = 90)	75 (83.3%)	6 (6.7%)	5 (5.6%)	1 (1.1%)	3 (3.3%)

Values are n (%). Grade IIIb and IVa are combined because of small individual cell counts (IIIb: n = 2; IVa: n = 2, one in each group). Descriptive presentation only; not subjected to formal statistical testing.

**Table 6:** Multivariate logistic regression — predictors of postoperative wound complications (SSI and/or seroma), full model.

Variable	OR	95% CI	*p*-value
Surgical technique: laparoscopic vs. open	0.59	0.26–1.33	0.203
Bowel resection: yes vs. no	0.57	0.06–5.47	0.630
Age (per 10 years)	0.97	0.72–1.29	0.810
Sex: male vs. female	0.67	0.29–1.55	0.344
BMI (per 1 kg/m^2^)	1.01	0.90–1.12	0.894
ASA score III–IV vs. I–II	1.41	0.61–3.24	0.421
Hernia type: incisional vs. inguinal	1.18	0.46–3.03	0.736
Hernia type: umbilical vs. inguinal	0.44	0.14–1.37	0.159
Hernia type: femoral vs. inguinal	0.52	0.10–2.63	0.427
Current smoker: yes vs. no	0.77	0.31–1.95	0.587
Contamination: yes vs. no	1.11	0.26–4.78	0.892
Symptom duration (per 10 h)	0.83	0.57–1.22	0.354

OR, odds ratio; CI, confidence interval. Model adjusted for age, sex, BMI, ASA class, hernia type, bowel resection, current smoking, symptom duration, and intraoperative contamination. Hosmer-Lemeshow goodness-of-fit *p* = 0.417. Nagelkerke R^2^ = 0.074. Overall likelihood-ratio *p* = 0.761. Events per variable ≈2.7.

**Table 7:** Reduced four-covariate sensitivity model — predictors of postoperative wound complications.

Variable	OR	95% CI	*p*-value
Surgical technique: laparoscopic vs. open	0.54	0.24–1.19	0.125
Bowel resection: yes vs. no	0.51	0.06–4.58	0.547
ASA score III–IV vs. I–II	1.27	0.58–2.79	0.543
Contamination: yes vs. no	1.36	0.34–5.48	0.662

OR, odds ratio; CI, confidence interval. Events per variable = 8. Overall likelihood-ratio p = 0.507. Nagelkerke R^2^ = 0.030.

**Table 8:** Covariate balance before and after propensity score matching.

Covariate	SMD before matching	SMD after matching
Age (years)	0.135	0.052
Sex (male)	0.090	0.059
BMI (kg/m^2^)	−0.072	0.082
ASA score III–IV	−0.114	0.091
Diabetes mellitus	0.052	0.101
Hypertension	−0.089	−0.059
COPD	0.031	−0.041
Coronary artery disease	−0.029	0.000
Current smoker	0.297	0.000
Symptom duration (hours)	0.012	0.026

SMD, standardized mean difference (laparoscopic minus open). |SMD|<0.10 is generally considered indicative of good covariate balance; all covariates met this threshold after matching except diabetes mellitus (0.101) and ASA score III–IV (0.091), which remained close to it.

**Table 9:** Outcomes in the propensity-score-matched cohort (68 matched pairs).

Outcome	Open (*n* = 68)	Laparoscopic (*n* = 68)	*p*-value
Length of hospital stay (days), mean ± SD	5.11 ± 0.88	3.96 ± 0.96	<0.001
Operation time (min), mean ± SD	75.9 ± 13.4	82.8 ± 16.3	0.020
Wound complication (SSI and/or seroma), *n* (%)	14 (20.6%)	11 (16.2%)	0.659
Postoperative ileus, *n* (%)	5 (7.4%)	1 (1.5%)	0.208
Surgical site infection, *n* (%)	7 (10.3%)	6 (8.8%)	1.000
Seroma, *n* (%)	8 (11.8%)	6 (8.8%)	0.779

Values are mean ± SD or *n* (%). Mann–Whitney U test for continuous variables; Fisher's exact test for categorical variables. SSI: surgical site infection.

**Table 10:** Subgroup analysis of primary outcomes by hernia type.

Hernia type	n (Open/Lap)	Wound comp. Open/Lap, %	LOS (days) Open/Lap	Op. time (min) Open/Lap	LOS p
Inguinal	39/37	20.5%/18.9% (*p* = 1.000)	5.23 ± 0.89/3.95 ± 0.82	76.2 ± 13.9/82.7 ± 18.1 (*p* = 0.076)	<0.001
Umbilical	21/25	14.3%/8.0% (*p* = 0.648)	4.92 ± 0.54/3.94 ± 1.01	77.4 ± 14.3/83.8 ± 16.9 (*p* = 0.290)	<0.001
Incisional	21/21	33.3%/14.3% (*p* = 0.277)	5.34 ± 0.84/4.10 ± 1.14	76.0 ± 11.9/82.4 ± 15.7 (*p* = 0.141)	<0.001
Femoral	9/7	22.2%/0.0% (*p* = 0.475)	5.32 ± 1.54/3.41 ± 0.70	72.1 ± 14.6/70.4 ± 6.2 (*p* = 0.916)	0.004

Values are % or mean ± SD; *p*-values from Fisher's exact test (wound complication) or Mann–Whitney U test [length of stay (LOS), operation time]. Postoperative ileus event counts were low in every subgroup (0–4 events) and are described in the text rather than tabulated.

**Table 11:** Sensitivity analyses restricted to mesh-used and no-bowel-resection subgroups.

Outcome	Open	Laparoscopic	*p*-value
Mesh-used subgroup (*n* = 141: 70 open, 71 lap.)			
Wound complication, *n* (%)	15 (21.4%)	10 (14.1%)	0.278
Length of hospital stay (days)	5.15 ± 0.89	3.88 ± 0.96	<0.001
Operation time (min)	75.6 ± 13.4	81.6 ± 16.3	0.057
Postoperative ileus, *n* (%)	6 (8.6%)	0 (0%)	0.013
No-bowel-resection subgroup (*n* = 171: 86 open, 85 lap.)			
Wound complication, *n* (%)	19 (22.1%)	12 (14.1%)	0.234
Length of hospital stay (days)	5.12 ± 0.82	3.84 ± 0.85	<0.001
Operation time (min)	75.0 ± 12.7	81.2 ± 16.7	0.027
Postoperative ileus, *n* (%)	6 (7.0%)	0 (0%)	0.029

Bowel resection was required in 9 patients overall (5.0%): 4 (4.4%) in the open group and 5 (5.6%) in the laparoscopic group, a difference that was not statistically significant (*p* = 1.000) (Table 3). Conversion from laparoscopic to open surgery occurred in 3 patients (3.3% of the laparoscopic group), most commonly due to inadequate visualization or bowel non-viability requiring resection.

Postoperative mortality, morbidity, and wound-related complications (surgical site infection and seroma) were assessed from inpatient records and outpatient follow-up. All patients were evaluated on postoperative day 10 for suture removal and clinical assessment, and complications were recorded based on clinical findings at that and subsequent visits, with a maximum Clavien-Dindo grade assigned per patient.

Postoperative ileus was defined *a priori* as the absence of flatus and/or bowel movement, together with persistent abdominal distension or intolerance of oral intake, beyond postoperative day 3, in the absence of a mechanical cause; cases meeting this definition were confirmed by the managing clinical team and, where obtained, supportive plain abdominal radiography. Readiness for discharge was defined by a standardized set of criteria applied prospectively during ward rounds: adequate oral intake tolerated for at least 24 h, passage of flatus or bowel movement, pain controlled on oral analgesia, absence of fever (>38 °C) for 24 h, and a clinically satisfactory wound at the time of review. All patients were additionally evaluated on postoperative day 10 for suture removal and clinical assessment, as previously stated; discharge itself, however, was determined by the above criteria rather than by a fixed postoperative day.

### 2.3 Statistical analysis

Statistical analysis was performed using Python (SciPy and statsmodels). Categorical variables were compared using Fisher's exact test, given the relatively small cell counts for several outcomes, and continuous variables were compared using the Mann–Whitney U test. Continuous variables are presented as mean ± standard deviation; because symptom duration and length of hospital stay showed right-skewed distributions on inspection (skewness 1.5–1.8), these two variables are additionally reported as median [interquartile range] for transparency, while age, BMI, and operative time were approximately symmetric (|skewness|≤0.6) and are reported as mean ± SD only. Categorical variables are expressed as frequency (percentage). A two-tailed *p*-value <0.05 was considered statistically significant.

Because surgical-site infection and seroma/hematoma were captured as two separate variables rather than as a single combined field, wound-related morbidity was defined *a priori* as a composite outcome (surgical site infection and/or seroma). To identify independent predictors of this composite outcome while accounting for baseline differences between groups, multivariate logistic regression was performed with age, sex, BMI, ASA class (I–II vs. III–IV), hernia type, current smoking status, symptom duration, intraoperative contamination, and the performance of bowel resection as covariates, with surgical technique (laparoscopic vs. open) as the primary predictor of interest. Model fit was assessed using the Hosmer-Lemeshow goodness-of-fit test and Nagelkerke R^2^. With 32 observed wound-complication events across this 12-covariate model, the events-per-variable ratio was approximately 2.7, below the commonly cited minimum of 10 for stable logistic regression; a reduced four-covariate model (surgical technique, bowel resection, ASA class, and intraoperative contamination; events-per-variable=8) was therefore fitted as a prespecified sensitivity analysis to confirm that the null finding for surgical technique was not an artifact of overfitting the larger model.

A *post-hoc* power analysis for the primary wound-complication comparison was performed using the observed effect size (Cohen's h = 0.23, based on rates of 22.2% in the open group and 13.3% in the laparoscopic group), a two-tailed alpha of 0.05, and 90 patients per group; the achieved power was approximately 0.35.

**Figure 4:**
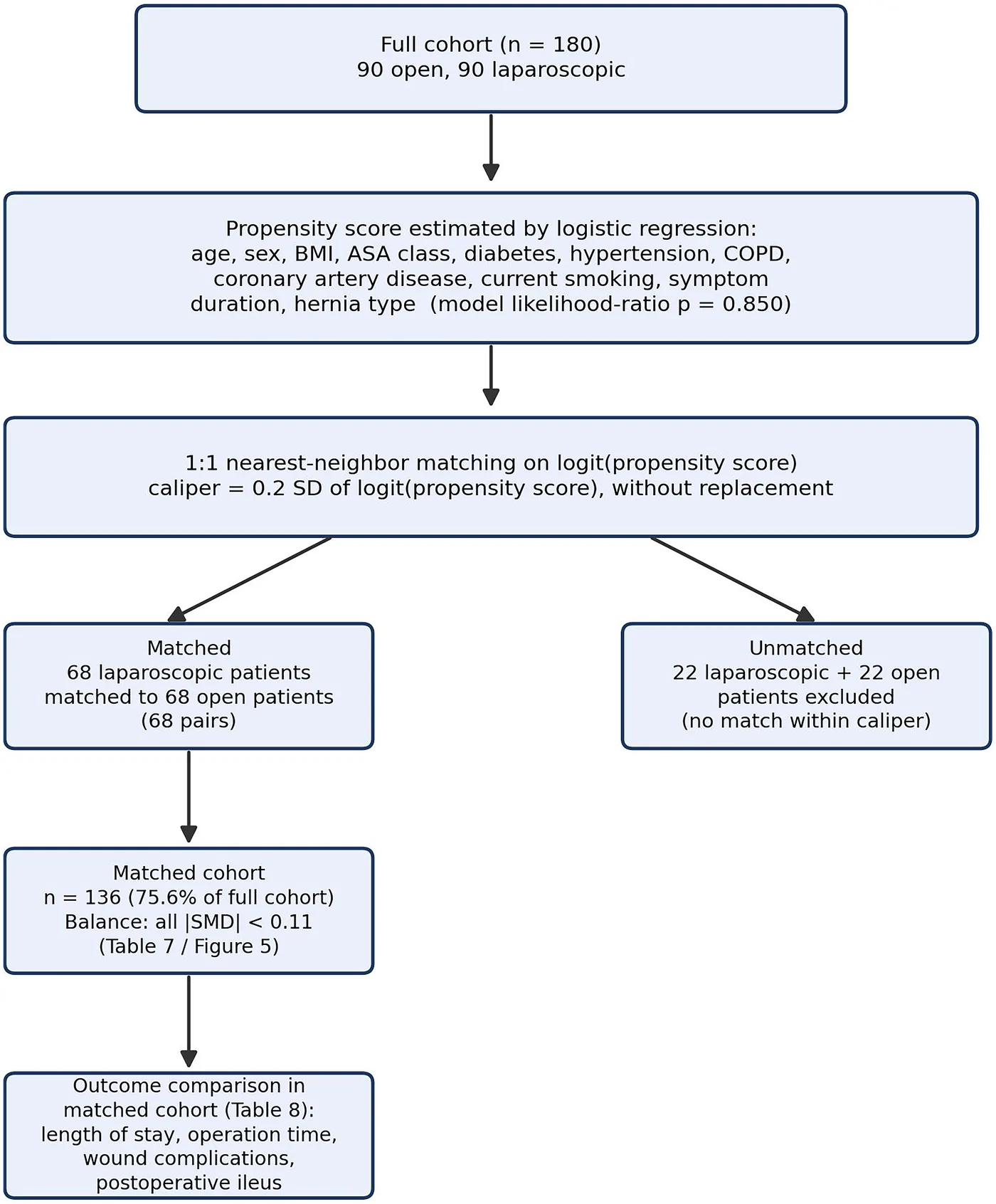
Propensity-score matching flow diagram, showing the propensity-score model, the 1:1 nearest-neighbor matching procedure (caliper 0.2 SD of the logit propensity score), and the resulting matched (*n* = 136, 68 pairs) and unmatched (*n* = 44) subsets.

**Figure 5:**
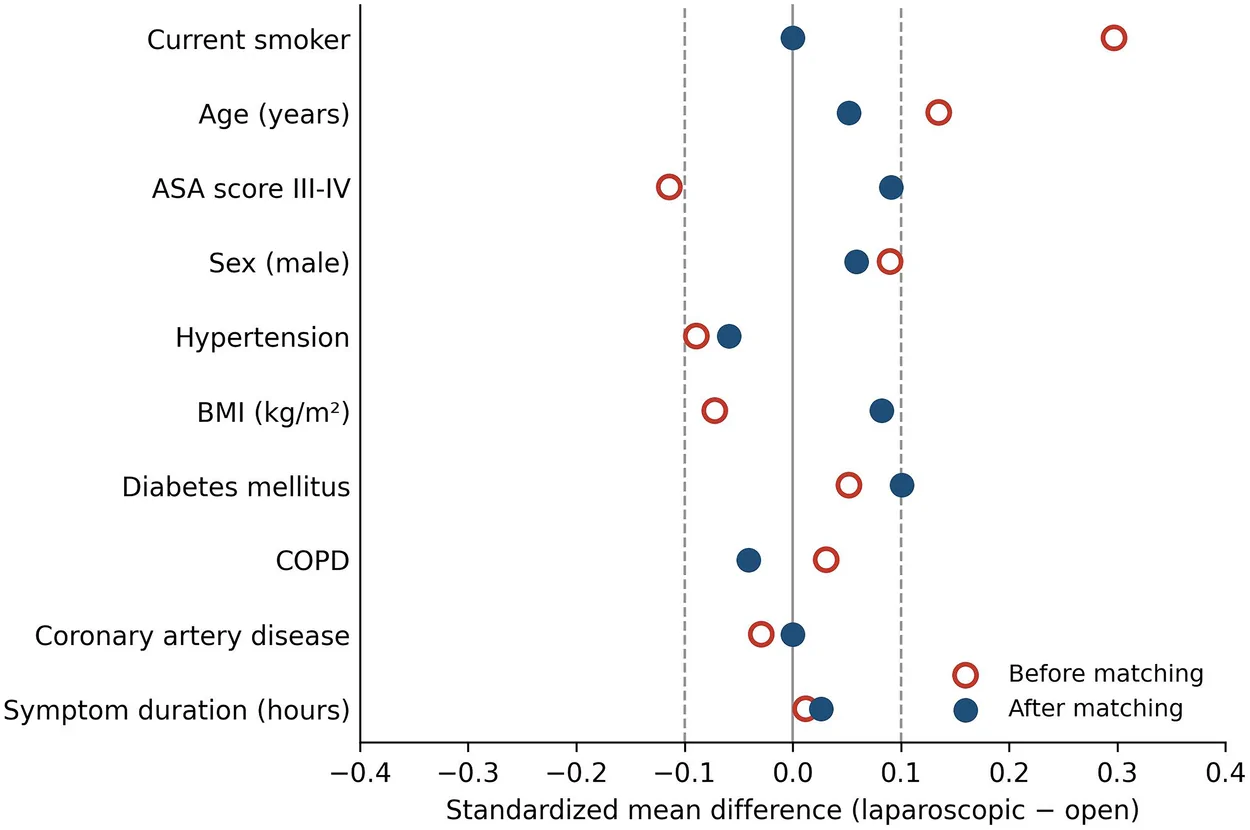
Love plot showing standardized mean differences (SMD) for each measured baseline covariate before and after propensity score matching. The dashed reference lines mark |SMD|=0.10, the threshold conventionally used to indicate good covariate balance.

Because surgical technique was not randomized, a propensity-score-matched sensitivity analysis was performed. A logistic regression model estimated the propensity to undergo laparoscopic (versus open) repair from age, sex, BMI, ASA class, diabetes, hypertension, COPD, coronary artery disease, current smoking, symptom duration, and hernia type. Patients were matched 1:1 without replacement using nearest-neighbor matching on the logit of the propensity score, with a caliper of 0.2 standard deviations of the logit propensity score; the matching workflow is summarized in Figure 4. Covariate balance before and after matching was assessed using standardized mean differences (SMD), with |SMD|<0.10 considered good balance, and is displayed graphically as a Love plot (Figure 5). Outcomes were compared in the matched cohort using the same statistical tests as in the overall cohort.

Prespecified subgroup analyses of the primary outcomes (wound complications, length of stay, operation time, postoperative ileus) were performed separately for each hernia type (inguinal, umbilical, incisional, femoral). Two additional sensitivity analyses were performed by restricting the cohort to (i) patients in whom mesh was used and (ii) patients who did not require bowel resection, to evaluate whether the main findings were driven by these subsets of patients. All hernia-type subgroup analyses and the two restricted-cohort sensitivity analyses, together with the propensity-matched analysis, were prespecified but exploratory; no formal statistical correction for multiple comparisons (e.g., Bonferroni or false-discovery-rate control) was applied across the more than a dozen significance tests generated for the wound-complication and ileus endpoints across the primary, matched, subgroup, and sensitivity analyses, and these secondary results should accordingly be interpreted as hypothesis-generating rather than confirmatory.

### 2.4 Ethics

Ethics committee approval was obtained from the institutional ethics committee (Approval No: 2026/51; Date: 04.02.2026). The study was conducted in accordance with the Declaration of Helsinki. Given the retrospective design, the requirement for informed consent was waived by the ethics committee.

## 3 Results

A total of 180 patients were included: 90 (50%) underwent open repair and 90 (50%) underwent laparoscopic repair. Baseline demographic and clinical characteristics are summarized in Table 1. The two groups were statistically similar in age, sex, BMI, diabetes, hypertension, COPD, coronary artery disease, smoking status, ASA class, and symptom duration before admission (all *p* > 0.05). Complete follow-up data were available for all 180 patients (100%); no patients were lost to follow-up during the study period.

Intraoperative findings and additional procedures are summarized in Table 3. Strangulation, contamination, and mesh use were comparable between groups. Conversion from laparoscopic to open approach occurred in 3 patients (3.3%) in the laparoscopic group and, by definition, in none of the open-group patients; this difference was not statistically significant (*p* = 0.246).

The “intraoperative bowel ischemic change” row (Table 3) reflects the intraoperative visual assessment at first inspection (serosal/subserosal discoloration), recorded in 15 patients overall (7 open, 8 laparoscopic). Of these, 10 patients (5 open, 5 laparoscopic) regained normal color and peristalsis after reduction and a period of reperfusion and were managed without resection, while 5 patients (2 open, 3 laparoscopic) underwent resection for unequivocal, non-viable transmural necrosis. A further 4 patients (2 open, 2 laparoscopic) underwent bowel resection for reasons other than necrosis at the initial visual assessment — specifically, bowel-wall injury sustained during reduction of a tightly incarcerated segment — bringing the total number of resections to 9 (4 open, 5 laparoscopic), as reported in Table 3 and Section 2.2.

Postoperative outcomes are summarized in Table 4. Operation time was significantly longer in the laparoscopic group (82.0 ± 16.7 min) than in the open group (76.0 ± 13.5 min, *p* = 0.025; Cohen's d = 0.39). Length of hospital stay was significantly shorter after laparoscopic repair [3.9 ± 1.0 days; median 3.8 (IQR 3.1–4.4)] than after open repair [5.2 ± 0.9 days; median 5.0 (IQR 4.6–5.5), *p* < 0.001; Cohen's d = −1.36]. Wound-related complications, defined as surgical site infection and/or seroma, occurred in 32 patients overall (17.8%): 12 (13.3%) in the laparoscopic group and 20 (22.2%) in the open group; although the rate was numerically lower after laparoscopy, this difference did not reach statistical significance (*p* = 0.172; risk difference −8.9 percentage points, 95% CI −20.0–2.2).

Considered individually, surgical site infection (6.7% vs. 13.3%, *p* = 0.213) and seroma (7.8% vs. 11.1%, *p* = 0.611) also did not differ significantly between groups. Postoperative ileus was significantly less frequent after laparoscopic repair (1.1%) than after open repair (8.9%, *p* = 0.034; risk difference −7.8 percentage points, 95% CI −14.0 to −1.5). Reoperation within 30 days (3.3% vs. 1.1%, *p* = 0.621) and hernia recurrence during follow-up (5.6% vs. 4.4%, *p* = 1.000) did not differ significantly between groups. No postoperative deaths occurred in either group (0/90 in each arm); the complete absence of mortality events precluded a formal statistical comparison of this outcome between techniques. Mean follow-up duration was comparable between groups (18.4 ± 5.2 months for laparoscopic vs. 18.9 ± 5.3 months for open, *p* = 0.754), with complete follow-up data (no losses) in both arms.

The distribution of maximum Clavien-Dindo complication grade by surgical technique is shown in Table 5. Grade ≥ II complications were numerically more frequent in the open group (21/90, 23.3%) than in the laparoscopic group (9/90, 10.0%); given the small number of higher-grade events in several categories, this distribution is presented descriptively rather than subjected to formal statistical testing.

### 3.1 Multivariable analysis of wound complications

On multivariate logistic regression for the composite wound-complication outcome, expanded to include current smoking, symptom duration, and intraoperative contamination alongside the previously specified covariates (Table 6), no covariate reached statistical significance, including surgical technique (laparoscopic vs. open: OR 0.59, 95% CI 0.26–1.33, *p* = 0.203) and bowel resection (OR 0.57, 95% CI 0.06–5.47, *p* = 0.630). Current smoking (OR 0.77, 95% CI 0.31–1.95, *p* = 0.587), symptom duration (per 10 h; OR 0.83, 95% CI 0.57–1.22, *p* = 0.354), and intraoperative contamination (OR 1.11, 95% CI 0.26–4.78, *p* = 0.892) were likewise not independently associated with wound complications, nor were age, sex, BMI, ASA class, or hernia type. The overall model did not significantly improve on the null model (likelihood-ratio *p* = 0.761), calibration was acceptable (Hosmer-Lemeshow *p* = 0.417), and the Nagelkerke R^2^ remained modest (0.074), indicating that the measured covariates — including the additional clinically motivated variables — explained only a small proportion of the variability in wound-complication risk.

Given the low events-per-variable ratio (≈2.7) for this 12-covariate model, a reduced, prespecified four-covariate sensitivity model (surgical technique, bowel resection, ASA class, and intraoperative contamination; events-per-variable=8) was also fitted (Table 7). This reduced model likewise identified no independent predictor of wound complications (technique OR 0.54, 95% CI 0.24–1.19, *p* = 0.125; bowel resection OR 0.51, 95% CI 0.06–4.58, *p* = 0.547; overall likelihood-ratio *p* = 0.507, Nagelkerke R^2^ = 0.030), confirming that the null finding for surgical technique in the full model is not simply an artifact of overfitting, and that the wide confidence intervals in Table 6 reflect genuine imprecision rather than a spuriously stabilized null result. A *post-hoc* power analysis based on the observed effect size for the primary wound-complication comparison (Cohen's h = 0.23) indicated an achieved power of approximately 35% at a two-tailed alpha of 0.05, suggesting the study was underpowered to detect a difference of this magnitude with confidence.

### 3.2 Propensity-score-matched sensitivity analysis

Because surgical technique was not randomized, a propensity-score model was fitted to estimate each patient's probability of undergoing laparoscopic (rather than open) repair from age, sex, BMI, ASA class, diabetes, hypertension, COPD, coronary artery disease, current smoking, symptom duration, and hernia type. This model was not significant overall (likelihood-ratio *p* = 0.850) and the only individually significant predictor of technique selection was current smoking (coefficient *p* = 0.038, favoring laparoscopy), which is consistent with the essentially unselected, balanced allocation of technique already shown in Table 1.

One-to-one nearest-neighbor matching on the logit propensity score (caliper 0.2 SD) yielded 68 matched pairs, comprising 136 of the 180 patients (75.6% of the cohort). Standardized mean differences (SMD) for all measured covariates fell below 0.11 after matching (Table 8, Figure 5), indicating good balance, compared with the already modest imbalances present before matching.

In the propensity-matched cohort (Table 9), length of hospital stay remained significantly shorter after laparoscopic repair (3.96 ± 0.96 days) than after open repair (5.11 ± 0.88 days, *p* < 0.001), and operation time remained significantly longer after laparoscopy (82.8 ± 16.3 min vs. 75.9 ± 13.4 min, *p* = 0.020), both consistent with the primary, unmatched analysis. However, the difference in postoperative ileus, which was statistically significant in the full cohort, was no longer significant in the smaller matched cohort (1/68, 1.5% laparoscopic vs. 5/68, 7.4% open, *p* = 0.208), and wound complications remained non-significantly lower after laparoscopy (11/68, 16.2% vs. 14/68, 20.6%, *p* = 0.659). Because the point estimates for ileus and wound complications remained directionally consistent with the primary analysis, this loss of significance most likely reflects reduced statistical power in the smaller matched sample (136 vs. 180 patients, a 24% reduction) rather than a qualitatively different effect of technique; however, given that no correction for multiple testing was applied across the primary, matched, subgroup, and sensitivity comparisons for this endpoint, a false-positive primary ileus finding cannot be formally excluded and this result should be read as hypothesis-generating.

### 3.3 Subgroup analysis by hernia type

**Figure 6:**
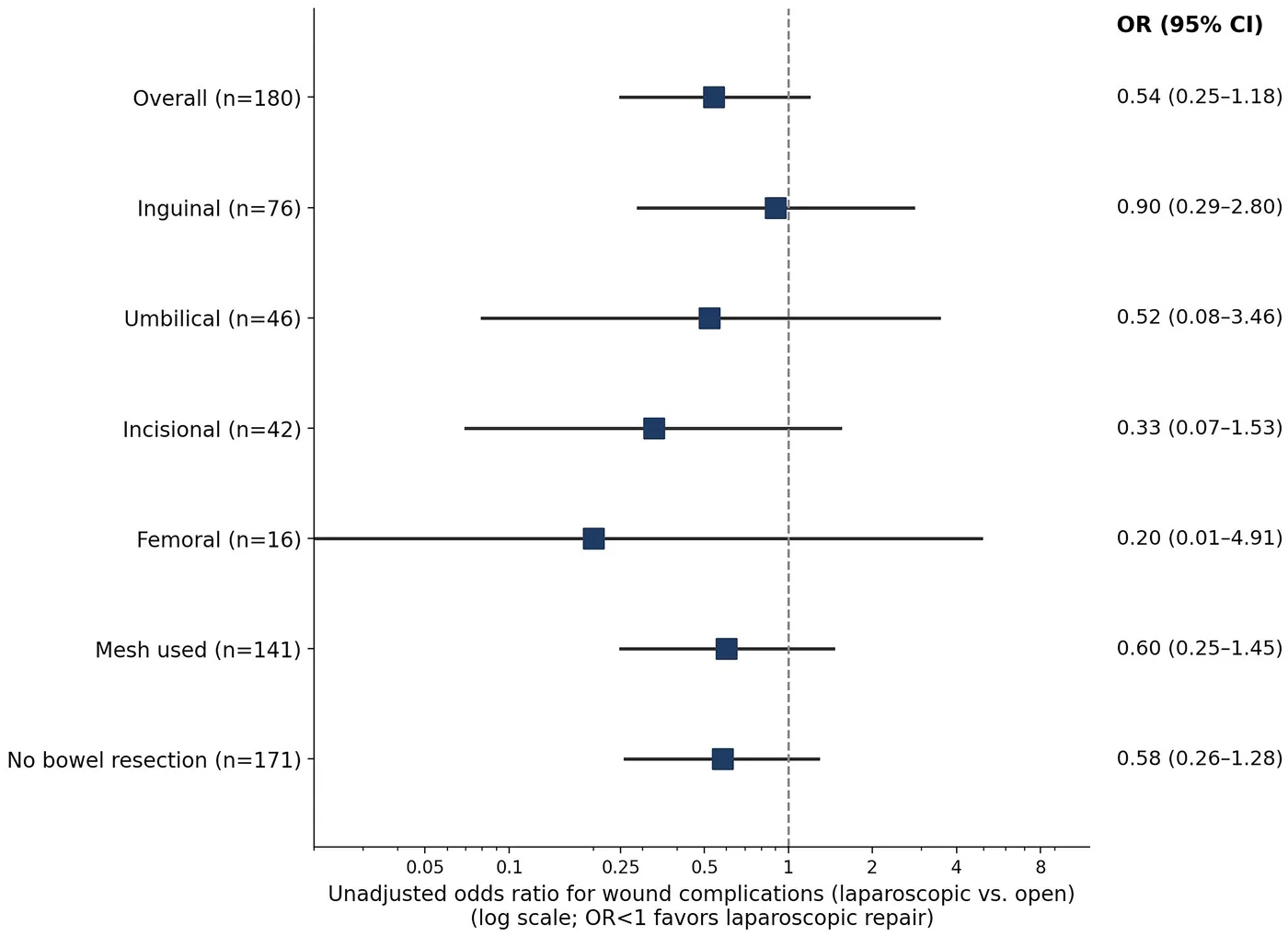
Forest plot of unadjusted odds ratios (with 95% confidence intervals) for the composite wound-complication outcome (surgical site infection and/or seroma), comparing laparoscopic with open repair, for the overall cohort, each hernia-type subgroup, and the mesh-used and no-bowel-resection sensitivity subgroups. These are unadjusted 2 × 2 comparisons and should not be confused with the covariate-adjusted odds ratios reported in Tables 6, 7. OR < 1 favors laparoscopic repair; the vertical dashed line denotes OR = 1 (no difference).

Prespecified subgroup analyses were performed separately for each hernia type (Table 10). These subgroup comparisons, like the sensitivity analyses in Section 3.6, were exploratory and not adjusted for multiple testing; the length-of-stay benefit associated with laparoscopic repair was consistent in direction and magnitude across all four hernia types and remained statistically significant within every subgroup despite the smaller subgroup sample sizes (all *p* ≤ 0.004). Unadjusted wound-complication odds ratios (laparoscopic vs. open) across hernia-type subgroups, together with the mesh-used and no-bowel-resection sensitivity subgroups, are displayed as a forest plot in Figure 6; all point estimates favored laparoscopic repair (OR < 1), and all 95% confidence intervals crossed 1, consistent with a directionally coherent but individually underpowered effect.

### 3.4 Sensitivity analyses: mesh-used and no-bowel-resection subgroups

When the analysis was restricted to the 141 patients in whom mesh was used (70 open, 71 laparoscopic), results were consistent with the overall cohort (Table 11): length of hospital stay remained significantly shorter (*p* < 0.001) and postoperative ileus significantly less frequent (*p* = 0.013) after laparoscopic repair, while operation time showed a similar but only borderline-significant trend toward being longer with laparoscopy (*p* = 0.057), and wound complications remained non-significantly lower after laparoscopy (*p* = 0.278).

Restricting the analysis to the 171 patients (86 open, 85 laparoscopic) who did not require bowel resection produced an essentially identical pattern, with significantly shorter hospital stay (*p* < 0.001), significantly longer operation time (*p* = 0.027), and significantly less postoperative ileus (*p* = 0.029) after laparoscopy, while wound complications remained non-significantly lower (*p* = 0.234). Together, these sensitivity analyses indicate that the principal findings of the study — a shorter hospital stay and a longer operation time with laparoscopic repair, and a lower rate of postoperative ileus — are not simply artifacts of the small number of patients requiring bowel resection or of patients in whom no mesh was placed.

## 4 Discussion

Laparoscopic hernia repair has gained increasing acceptance over the past decade in both elective and emergency settings. Numerous contemporary studies and meta-analyses have shown that minimally invasive techniques are associated with reduced postoperative pain, lower wound complication rates, and shorter hospital stay compared with open repair (10–12, 16).

In the present cohort, laparoscopic repair was associated with a significantly shorter hospital stay and a significantly lower rate of postoperative ileus than open repair, consistent with prior reports linking smaller incisions and reduced tissue trauma to faster bowel recovery and earlier discharge (17–20). Wound-related complications — surgical site infection and seroma, considered individually or as a composite outcome — were numerically less frequent after laparoscopy (13.3% vs. 22.2%; risk difference −8.9 percentage points, 95% CI −20.0–2.2) but this difference did not reach statistical significance, and multivariate adjustment — in both the full model and a reduced, prespecified four-covariate sensitivity model — did not identify surgical technique as an independent predictor of wound complications. A *post-hoc* power analysis indicated that, given the observed effect size and the achieved sample size, the study had only approximately 35% power to detect this difference; a true benefit of laparoscopy on wound morbidity therefore cannot be excluded on the basis of these data alone, but neither can the possibility that no true difference exists.

No postoperative deaths occurred in either group. While reassuring, this finding should not be over-interpreted: with zero events among 180 patients, the study had no ability to formally compare mortality between techniques, and the true difference in mortality risk, if any, remains undetermined by this cohort.

Operation time was, on average, approximately six minutes longer in the laparoscopic group than in the open group (Cohen's d = 0.39, a small-to-moderate effect). This may reflect the additional steps involved in establishing pneumoperitoneum, achieving adequate visualization of the hernia orifice, and secure intraperitoneal mesh fixation, and is consistent with prior reports of a modest time penalty associated with laparoscopic emergency hernia repair, particularly during the learning curve (21). By contrast, the length-of-stay difference was large in magnitude (Cohen's d = −1.36), suggesting this is the more clinically substantial of the two significant continuous-outcome differences.

Unlike some previously published single-center series in this population, the two treatment groups in the present cohort were of equal size (90 open vs. 90 laparoscopic) and closely balanced across all measured baseline characteristics, including symptom duration before admission. This balance strengthens confidence that the observed differences in length of stay and postoperative ileus are not simply artifacts of a small, highly selected laparoscopic subgroup, although allocation of technique was still made by the surgical team rather than randomized, and unmeasured selection factors — such as intraoperative judgment about bowel viability — cannot be excluded.

Because baseline covariates were already balanced before matching (Table 1, all *p* > 0.05) and the propensity model itself was not statistically significant (likelihood-ratio *p* = 0.850), propensity-score matching did not correct any detectable measured imbalance in this cohort; its purpose here was as a robustness check against non-randomized allocation and residual confounding correlated with the measured covariates, rather than as a bias-correction step for an observed imbalance. This robustness check came at a genuine power cost — a 24% reduction in sample size (180→136) — which is the most plausible explanation for why the postoperative ileus difference lost significance in the matched cohort, and readers should weigh this trade-off when interpreting the matched-cohort results alongside the primary, full-cohort analysis.

The robustness of these findings was further supported by several additional analyses. A propensity-score model predicting technique selection from baseline characteristics was itself not statistically significant (likelihood-ratio *p* = 0.850), reinforcing the impression from Table 1 that allocation to laparoscopy versus open surgery was not strongly driven by measurable patient factors in this cohort. Propensity-score matching nonetheless improved covariate balance further (all |SMD|<0.11) and, in the resulting 68 matched pairs, reproduced the significantly shorter hospital stay and significantly longer operation time seen in the overall cohort. The loss of statistical significance for postoperative ileus in the matched analysis (*p* = 0.208, versus *p* = 0.034 in the full cohort) is most parsimoniously explained by the roughly 24% reduction in sample size that matching entailed rather than by a qualitative change in the underlying effect, since the point estimate remained in the same direction (7.4% open vs. 1.5% laparoscopic). Subgroup analyses stratified by hernia type showed a remarkably consistent length-of-stay advantage for laparoscopy across inguinal, umbilical, incisional, and femoral hernias (all *p* ≤ 0.004), arguing against the possibility that this finding is confined to, or driven by, a single hernia subtype. Wound-complication rates were directionally lower after laparoscopy in every hernia-type subgroup (most notably in incisional hernias, 14.3% vs. 33.3%) without reaching significance in any individual subgroup, again consistent with an underpowered but directionally coherent signal. Sensitivity analyses restricted to patients in whom mesh was used and to patients who did not require bowel resection reproduced the same overall pattern — shorter stay, longer operation time, less ileus, and a non-significant trend toward fewer wound complications with laparoscopy — indicating that these findings are not attributable to the small number of mesh-omitted or bowel-resection cases. Finally, both the full multivariate model (12 covariates) and a reduced four-covariate sensitivity model reached the same substantive conclusion: none of the assessed factors, including surgical technique, was independently associated with wound complications, and model calibration remained acceptable in both (Hosmer-Lemeshow *p* = 0.417 for the full model).

### 4.1 Limitations

Several limitations warrant emphasis. First, this remains a retrospective, single-center study, and the choice between open and laparoscopic repair was not randomized but was determined by the surgical team based on clinical judgment, hemodynamic stability, and suspected bowel compromise; unmeasured confounding related to this allocation process cannot be excluded even though the measured baseline characteristics were well balanced between groups. Second, several outcomes of interest — bowel resection (9 patients), conversion to open surgery (3 patients), reoperation within 30 days (4 patients), and postoperative mortality (0 patients) — were rare events, which limits statistical power for these comparisons and widens the confidence intervals around the corresponding effect estimates; the wide 95% confidence interval around the odds ratio for bowel resection in the multivariate model (0.06–5.47) illustrates this directly, and the reduced four-covariate sensitivity model (95% CI 0.06–4.58) shows a similarly wide interval despite improved events-per-variable. Third, although the composite wound-complication outcome (surgical site infection and/or seroma) is a pragmatic proxy for the surgical-site morbidity described in earlier single-center series, it does not distinguish infection from sterile fluid collection, and the underlying biological mechanisms and management implications of these two complications can differ. Fourth, the *post-hoc* power analysis indicates that the study was underpowered (approximately 35% power) to reliably detect the observed difference in wound complications between techniques, so this non-significant result should not be interpreted as evidence of equivalence between approaches. Fifth, the propensity-score-matched analysis, while reassuring, matched only 68 of 90 laparoscopic patients (75.6% of the cohort); the 44 unmatched patients were disproportionately at the extremes of the propensity-score distribution, and results from the matched cohort may not fully generalize to these excluded patients. Sixth, heterogeneity in hernia type (inguinal, incisional, umbilical, and femoral) may have introduced additional variability in outcomes; although hernia type was not a significant independent predictor of wound complications in our model, and the length-of-stay benefit of laparoscopy was consistent across all four subgroups, the individual hernia-type subgroups — particularly femoral hernia (*n* = 16) — were too small to exclude residual confounding or to detect subgroup-specific differences in wound complications with confidence. Finally, surgical expertise, institutional resources, and the availability of surgeons experienced in laparoscopic emergency repair are likely to vary substantially across centers, and the operative-time and conversion-rate findings in particular may not generalize to institutions earlier in their laparoscopic emergency-surgery learning curve.

The sample size of this study (*n* = 180, with equal-sized open and laparoscopic groups) is larger than that of many previously published single-center series on emergency hernia repair, and the two groups were well balanced on all measured baseline characteristics. Nevertheless, prospective, ideally multicenter, studies employing predefined selection criteria for laparoscopy — and adequately powered for the wound-complication and mortality endpoints — are needed to determine whether the numerically lower wound-complication rate observed after laparoscopy in this cohort reflects a true treatment effect (22, 23).

Despite these limitations, our findings are consistent with the broader literature suggesting that laparoscopic repair is associated with faster postoperative recovery — shorter hospital stay and a lower rate of ileus — in patients undergoing emergency surgery for incarcerated or strangulated hernia, while the effect of technique on wound-related morbidity remains statistically inconclusive in this cohort (24–27).

## 5 Conclusions

In this retrospective single-center cohort of 180 patients undergoing emergency repair of incarcerated or strangulated abdominal wall hernia, laparoscopic repair was associated with a significantly shorter hospital stay and a significantly lower rate of postoperative ileus than open repair, but with a longer operation time. Wound-related complications (surgical site infection and/or seroma) were numerically, but not statistically significantly, less frequent after laparoscopy, and both a full and a reduced-covariate multivariate model did not identify surgical technique as an independent predictor of wound complications; a *post-hoc* power analysis suggests the study was underpowered for this comparison (achieved power ≈35%).

A propensity-score-matched analysis, prespecified subgroup analyses by hernia type, and sensitivity analyses restricted to mesh-used and no-bowel-resection patients all reproduced the shorter hospital stay, longer operation time, and non-significantly lower wound-complication rate seen in the primary analysis, supporting the robustness of these findings across analytic approaches, although the ileus benefit did not remain significant in the smaller matched cohort and none of these secondary comparisons was adjusted for multiple testing. No postoperative deaths occurred in either group, precluding formal comparison of mortality. Because treatment allocation was not randomized, these associations should be interpreted with caution and cannot be assumed to reflect a causal effect of technique alone. Laparoscopic repair may represent a reasonable option in carefully selected, hemodynamically stable patients with incarcerated or strangulated hernia, but adequately powered, prospective multicenter studies using predefined selection criteria are needed before firm treatment recommendations can be made regarding wound-related morbidity and mortality.

## Data Availability

The original contributions presented in the study are included in the article/Supplementary Material, further inquiries can be directed to the corresponding author.

## Supplementary material

Supplementary File 1 Completed STROBE checklist for cohort studies.
