# Supplementary material for: Laparoscopic versus open emergency repair for strangulated abdominal wall hernias: a propensity-score-matched retrospective cohort study
Source: Front Surg. 2026 Sep 9;13:1949053. doi: 10.3389/fsurg.2026.1949053 (PMC13597863; doi:10.3389/fsurg.2026.1949053)
Supplement: Supplementary file 1 [file Supplementaryfile1.docx]

**STROBE Statement—Checklist of items that should be included in reports of cohort studies**

**Manuscript ID: 1949053**

*Laparoscopic versus open emergency repair of incarcerated or strangulated abdominal wall hernias*

| **Section** | **Item** | **Recommendation** | **Reported in manuscript** |
| --- | --- | --- | --- |
| Title and abstract | 1 | (a) Indicate the study’s design with a commonly used term in the title or abstract. | Title/Abstract |
| Title and abstract | 1 | (b) Provide in the abstract an informative and balanced summary of what was done and what was found. | Abstract |
| Background/rationale | 2 | Explain the scientific background and rationale for the investigation being reported. | Introduction |
| Objectives | 3 | State specific objectives, including any prespecified hypotheses. | Introduction |
| Study design | 4 | Present key elements of study design early in the paper. | Methods, Study design |
| Setting | 5 | Describe the setting, locations, and relevant dates, including periods of recruitment, exposure, follow-up, and data collection. | Methods |
| Participants | 6 | Give eligibility criteria and the sources and methods of selection of participants. Describe methods of follow-up. | Methods; Figure 1 |
| Variables | 7 | Clearly define all outcomes, exposures, predictors, potential confounders, and effect modifiers. Give diagnostic criteria, if applicable. | Methods |
| Data sources/measurement | 8 | For each variable of interest, give sources of data and details of methods of assessment. Describe comparability of assessment methods if there is more than one group. | Methods |
| Bias | 9 | Describe any efforts to address potential sources of bias. | Methods; propensity-score sensitivity analysis |
| Study size | 10 | Explain how the study size was arrived at. | Methods; 180 eligible patients, 90 per group |
| Quantitative variables | 11 | Explain how quantitative variables were handled in the analyses. If applicable, describe which groupings were chosen and why. | Statistical analysis |
| Statistical methods | 12(a) | Describe all statistical methods, including those used to control for confounding. | Statistical analysis |
| Statistical methods | 12(b) | Describe any methods used to examine subgroups and interactions. | Statistical analysis; subgroup analyses |
| Statistical methods | 12(c) | Explain how missing data were addressed. | Results; complete follow-up/no losses reported |
| Statistical methods | 12(d) | If applicable, explain how loss to follow-up was addressed. | Results; no losses to follow-up |
| Statistical methods | 12(e) | Describe any sensitivity analyses. | Methods/Results; reduced model, PSM, mesh-used and no-bowel-resection analyses |
| Participants | 13(a) | Report numbers of individuals at each stage of study—potentially eligible, examined for eligibility, confirmed eligible, included, completing follow-up, and analysed. | Results; Figure 1 |
| Participants | 13(b) | Give reasons for non-participation at each stage. | Figure 1 |
| Participants | 13(c) | Consider use of a flow diagram. | Figure 1 |
| Descriptive data | 14(a) | Give characteristics of study participants and information on exposures and potential confounders. | Table 1 |
| Descriptive data | 14(b) | Indicate number of participants with missing data for each variable of interest. | Results; no missing follow-up reported |
| Descriptive data | 14(c) | Summarise follow-up time. | Table 4; mean follow-up ~18 months |
| Outcome data | 15 | Report numbers of outcome events or summary measures over time. | Tables 3–5 |
| Main results | 16(a) | Give unadjusted estimates and, if applicable, confounder-adjusted estimates and their precision. Make clear which confounders were adjusted for and why. | Tables 4, 6, 7 |
| Main results | 16(b) | Report category boundaries when continuous variables were categorized. | Methods/Tables where applicable |
| Main results | 16(c) | If relevant, consider translating estimates of relative risk into absolute risk for a meaningful time period. | Results; absolute risk differences reported for wound complications and ileus |
| Other analyses | 17 | Report other analyses done—e.g., analyses of subgroups and interactions, and sensitivity analyses. | Tables 8–11; Figures 4–6 |
| Key results | 18 | Summarise key results with reference to study objectives. | Discussion |
| Limitations | 19 | Discuss limitations of the study, taking into account sources of potential bias or imprecision. | Discussion, Limitations |
| Interpretation | 20 | Give a cautious overall interpretation of results considering objectives, limitations, multiplicity of analyses, results from similar studies, and other relevant evidence. | Discussion/Conclusions |
| Generalisability | 21 | Discuss the generalisability (external validity) of the study results. | Discussion, Limitations |
| Funding | 22 | Give the source of funding and the role of the funders for the present study and, if applicable, for the original study on which the present article is based. | Funding: no financial support received |
